# Deep Sensing: Inertial and Ambient Sensing for Activity Context Recognition Using Deep Convolutional Neural Networks

**DOI:** 10.3390/s20133803

**Published:** 2020-07-07

**Authors:** Abayomi Otebolaku, Timibloudi Enamamu, Ali Alfoudi, Augustine Ikpehai, Jims Marchang, Gyu Myoung Lee

**Affiliations:** 1 Department of Computing, Sheffield Hallam University, Sheffield S1 2NU, UK; t.enamamu@shu.ac.uk (T.E.); jims.marchang@shu.ac.uk (J.M.); 2 College of Computer Science & Information Technology, University of Al-Qadisiyah, Al Diwaniyah 58002, Iraq; a.s.alfoudi@qu.edu.iq; 3 Department of Engineering and Mathematics, Sheffield Hallam University, Sheffield S1 2NU, UK; a.ikpehai@shu.ac.uk; 4 Department of Computer Science, Liverpool John Moores University, Liverpool L3 3AF, UK; g.m.lee@ljmu.ac.uk

**Keywords:** activity context sensing, smartphones, deep convolutional neural networks, smart devices

## Abstract

With the widespread use of embedded sensing capabilities of mobile devices, there has been unprecedented development of context-aware solutions. This allows the proliferation of various intelligent applications, such as those for remote health and lifestyle monitoring, intelligent personalized services, etc. However, activity context recognition based on multivariate time series signals obtained from mobile devices in unconstrained conditions is naturally prone to imbalance class problems. This means that recognition models tend to predict classes with the majority number of samples whilst ignoring classes with the least number of samples, resulting in poor generalization. To address this problem, we propose augmentation of the time series signals from inertial sensors with signals from ambient sensing to train Deep Convolutional Neural Network (DCNNs) models. DCNNs provide the characteristics that capture local dependency and scale invariance of these combined sensor signals. Consequently, we developed a DCNN model using only inertial sensor signals and then developed another model that combined signals from both inertial and ambient sensors aiming to investigate the class imbalance problem by improving the performance of the recognition model. Evaluation and analysis of the proposed system using data with imbalanced classes show that the system achieved better recognition accuracy when data from inertial sensors are combined with those from ambient sensors, such as environmental noise level and illumination, with an overall improvement of 5.3% accuracy.

## 1. Introduction

According to the World Health Organization (WHO), insufficient physical activity is one of the leading risk factors for death worldwide [1]. This could lead to non-communicable illnesses, such as cardiovascular diseases, cancer, diabetes, and many more. Physical activity is defined as “any bodily movement produced by skeletal muscles that require energy expenditure, including activities undertaken while working, carrying out household chores, traveling, and engaging in recreational pursuits” [1]. To improve the physical wellbeing of people and to reduce the pressure on health infrastructure and the cost of healthcare delivery, governments now encourage people to engage in various forms of physical activities. In this regard, various research works have been conducted to provide solutions that support physical activities. Besides, being able to predict or recognize user activity contexts is not only important in health monitoring applications, but such information can also be used in designing and implementing other intelligent applications in transportation, security, and intelligent recommendation systems, etc. [2,3,4,5]. This is inevitable because recent advances in ubiquitous computing, cloud computing, Artificial Intelligence (AI), and developments in network solutions, such as the 5G, etc., open up greater opportunities. Today, many people not only use smart devices that have the incredible capability for sensing human activities and contexts, but these devices can also provide solutions that promote and improve human general wellbeing. Besides, smart objects are everywhere, interacting with our living spaces and producing an incredible amount of data [2]. Exploiting this data for developing more intelligent applications has seen keen academic and industrial interests [2,4]. One of the key research interests is how to use the data to identify meaningful information not only about mobile users but also in the environments. In particular, researchers in the last decade have investigated various approaches for recognizing human activity contexts by collecting a large volume of data from body-worn devices or smartphones, as well as other sensory devices, to develop automated solutions using various AI techniques [2,6,7,8].

Activity context recognition is one of the techniques that has been widely used to study human behaviors, such as walking, running, driving, eating, jogging, running patterns, etc. [2,3,4]. With a better understanding of the patterns of these behaviors, more intelligent applications in the domain of mobile healthcare systems, information systems, such as service recommendation systems, etc., are now a reality [3,4]. However, recognizing activity context despite the impressive efforts and results by enthusiastic researchers still has some significant challenges. One of such challenges, which has not been adequately addressed is that of class imbalance [2]. It is common with some human activities involving human behavioral monitoring. For example, some activities occur more frequently, e.g., sleeping, while others occur infrequently, e.g., climbing stairs. This problem is particularly common with sensing in unconstrained environments. 

**Figure 1:**
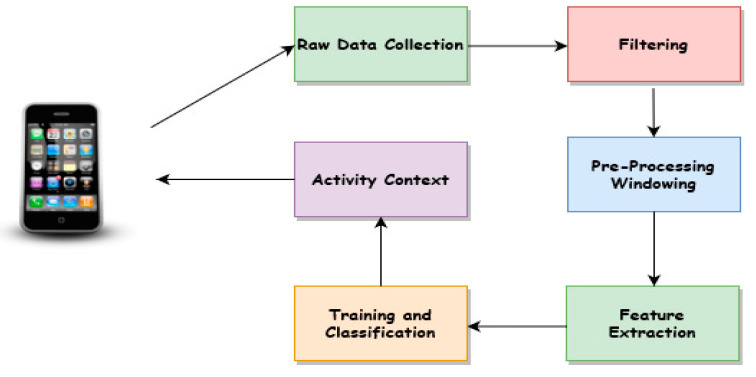
Classical activity context recognition processes.

Another key challenge is that current approaches in the realm of human activity context recognition have largely focused on identifying individual activity by using handcrafted approaches to extract useful features from the collected data [9]. Feature extraction is one of the crucial steps in activity context recognition that captures information, which discriminates various activity contexts [2,3,4,10]. In our previous work, we reported extensively on the traditional approaches used in activity context recognition applications [3]. The traditional activity context recognition system as depicted in Figure 1 consists of key processing steps, including data collection, data filtering, data pre-processing, such as segmentation, handcrafted features extraction, model training, and activity context classification. Since this classical technique relies on handcrafted feature extraction, it is prone to recognition errors and cannot generalize.

Recently, deep learning algorithms have achieved unparalleled performance in several areas, such as image processing, visual object recognition, natural language processing, driverless cars, robots, etc. [6,7,8,9,10,11]. DCNNs are now widely used for the development of automatic human activity context recognition [6,7,8,9,12,13,14,15,16,17,18]. Representational learning of activity context from raw sensor data using a DCNN has been proposed for automatic feature extraction in activity context recognition [12,13,14,15,16,17,18,19,20,21]. Besides, deep learning algorithms have the capability for unsupervised and incremental learning because of its deep network structure compared to the traditional neural network. A DCNN is composed of multiple building blocks, such as convolutional layers, pooling layers, and fully connected layers [9,13,14,15,16,17]. It has been designed to automatically and adaptively learn spatial hierarchies of features, from low to high-level patterns, through backpropagation algorithm [16,19,20,21,22]. Its attraction is due to its special architecture with a strong ability to learn filters and apply them to small-sub regions of data. This unsupervised feature learning, which is performed in the convolution layers, allows them to easily capture hidden local patterns and variations in the data. The resulting feature map is then passed to the fully connected layers for activity context classification. The convolutional layers are trained alongside other layers of the network as their outputs serve as the inputs of other convolutional layers. The convolutional operation exploits effectively the local temporal dependency of time series data, while its pooling operation cancels the impact of small translation of the input. With its weight sharing feature, the convolution operation of the DCNN allows reservation of scale invariance, which, in activity context recognition, can discriminate between two similar or identical classes. Furthermore, this operation helps to capture local dependencies of the signals [9]. For example, it would be able to capture the dependencies between inertial sensing signals and those of nearby ambient sensors. It also lowers the computational cost by reducing the number of connections between convolutional layers [6,9,11]. With the capability to be optimized using backpropagation, it is an excellent deep learning architecture that produces minimal prediction error [22].

Most research works using DCNNs have focused on using visual data from video cameras [21] or inertial sensors, such as accelerometers, and gyroscopes [9,11,15,16]. Ambient sensing has been largely ignored, however, ambient sensing is used to capture interactions between humans and the environment. Belapurka et al. [23] made a strong case for using ambient sensing for recognizing human activity contexts. However, they only proposed it as a means of tackling privacy-related problems of human activity context recognition. Ambient sensors are usually embedded in the environment and examples include temperature, light, sound, pressures sensors, etc. But modern mobile devices, such as smartphones, have these sensors, and they are important sources of data that could be explored to improve the performance of human activity recognition models. To provide richer contextual information and address class imbalance challenge of activity context recognition, we proposed to enrich the traditional inertial dataset with ambient sensing by using the CNN for automatic feature extraction to improve both the local and global performance of models with imbalanced classes.

The key contribution of this article is threefold:

(1) We demonstrate that with inertial and ambient sensors, namely environment noise level and illumination could improve recognition performance using data with imbalanced classes.
(2) We performed extensive hyperparameter tuning to select optimal values to build the DCNN model.
(3) We demonstrate that the DCNN can perform better recognition with raw sensing data without handcrafted features than with manually extracted features.

The rest of the paper is organized as follows. Section 2 presents relevant related work. In Section 3, we present details of the proposed system for classifying context from raw sensor data. Section 4 presents our experiments and evaluation results. In Section 5, we conclude and outline our future work.

## 2. Related Work

Human activity contexts are important contextual information, especially in the new ubiquitous computing environments. This type of contextual information will play an important role in our daily lives through various intelligent applications. Human activity contexts coupled with the emergence of the Internet of Things (IoT) as the de facto means of gathering huge volumes of data relating to the human environment and their behaviors is revolutionizing how we engineer intelligent systems. In addition, the new paradigm of emerging IoT network infrastructure enables billions of interconnected devices to communicate and exchange information and is the future platform for providing intelligent applications in various domains, such as health and wellbeing monitoring systems, enhanced retail recommendation applications, smart homes, and smart cities. To engineer such systems, there is a need to provide an automatic way of recognizing and classifying human activity context. The process for automatic recognition of human activity context is generally known as Human Activity Recognition (HAR) [15,16,17]. This is a typical pattern recognition problem based on using traditional algorithms, such as support vector machines, K-Nearest Neighbor, naïve Bayes, decision trees, random forest, etc. [3,4,10,24].

Research activities in the human activity context can be broadly categorized into two. Video-based human activity recognition and sensor-based activity recognition [4,7,8,9,10,11,12,13,14,15,16,17,18]. The sensor-based activity recognition process focused more on using data generated by inertial sensors, such as accelerometer and gyroscope, for recognizing human locomotive activities by either placing these sensors on various parts of the human body or using smartphones [16,24,25,26]. The video-based human activity recognition has focused on using video surveillance data in the activity recognition processes [21]. In recent years, many research works have explored various algorithms, whilst building new ones, to automatically identify human activities. The conventional machine learning algorithms have been extensively explored and widely reported in the literature [2,3,4,10,16]. For example, in our previous work, we explored various traditional classification algorithms for automatic context recognition [3]. The result was applied in the development of a context model for an intelligent context-aware recommendation system. Other works based on classical machine learning algorithms and handcrafted feature extraction processes have been extensively reported [4,24,25,26,27]. For example, authors in Reference [18] proposed a new approach using a descriptor-based approach to human activity recognition. They handcrafted time and frequency domain features from accelerometer and gyroscope signals and then used conventional support vector machines and k-nearest neighbor algorithms. In Reference [28], Straczkiewicz and Onnela provide a comprehensive review of several human activity recognition research works using classical machine learning algorithms. The majority of the reported works using traditional machine learning algorithms are based on handcrafted feature extraction processes. Zeng et al. [9] report that, although these works might have demonstrated good performance recognizing one activity, they, however, perform poorly recognizing others due to class imbalance. They also noted that these works cannot capture local dependencies of an activity signal, as well as not being able to preserve scale invariance. This explains why some models struggle to discriminate between jogging and running contexts [25]. 

In recent years, several works also focused on using deep neural networks for activity recognition using signals from only inertial sensors [5,6,7,8,9,12,13,14,15,16,17,18,19,29,30]. This new development is due to the incredible advancements in compute power. For example, one of the earliest works is the one presented by Jiang and Yin [12]; in their paper, rather than exploring handcrafted features from time-series sensor signals, they assembled signal sequences of accelerometers and gyroscopes into a novel activity image and used the data to train the DCNN to automatically learn the optimal features for the activity recognition task. Another important and interesting work is the one presented by Zeng et al. [9]. They also developed a system that automatically extracts features from raw sensing data, using a CNN with partial weight sharing technique. Another interesting work is the one presented by Zebin et al. [29], where signals from inertial sensors have been used to train the DCNN for automatic feature extraction and activity recognition.

Some works combine statistical features with deep learning to automatically recognize human activities. For example, Hassan et al. [15] present a robust human activity recognition system using smartphone sensors and deep learning algorithms. In that work, from gyroscope and accelerometer data, they extracted statistical features, such as mean, median, autoregressive coefficients, etc., which are then fed into the DCNN. Similarly, Ignatov [16] proposed a deep neural network architecture that combines shallow DCNN for unsupervised feature extraction together with statistical features to encode global characteristics of smartphone sensor data. Ronao and Cho [17] proposed a DCNN-based recognition system where they fed raw inertial sensor data into the DCNN model for automatic feature extraction. To improve the performance of the model, they combined manually extracted fast Fourier Transform of the HAR dataset.

Researchers have also come up with an innovative way to identify human activity context using data from sensors other than the traditional inertial or motion sensors to augment these classical sensing data. The rationale is to address the problems, such as class imbalance, associated with using inertial sensors to improve activity context recognition performance. Researchers, such as Belapurka et al. [23], made a very strong case for using ambient sensing for recognizing human activity contexts. However, they only proposed it as a means of tackling privacy-related problems of human activity context recognition. Some others also used ambient sensing to address the computational complexity, power consumption, cost and recognition accuracy, or poor generalization issues. An example of such work is the one presented by Golestani and Moghaddam [31], where they introduced magnetic induction-based human activity recognition to effectively detect physical movements using magnetic induction signals rather than inertial sensors signals. They compared the performance of their work using traditional machine learning algorithms, such as Support Vector Machines (SVM), K-Nearest Neighbor ( KNN), etc., with deep learning algorithms, such as deep long short-term memory (LSTM), and concluded that deep learning outperformed the traditional algorithms.

In terms of combining ambient sensing data with inertial sensing data for activity recognition, only a few works have reported this approach. One of the latest reports is the one conducted by Cruciani et al. [13], in which they used audio and inertial datasets to pre-train a DCNN model for automatic human activity recognition. Another recent work is presented by Schrader et al. [32], which uses audio signals and cameras as ambient sensors in addition to other sensors to recognize elderly people’s activities for rehabilitation and early intervention. We proposed a combination of 3 inertial sensors, namely accelerometer, gyroscope, and magnetometer. This data is combined with ambient sensing data from environmental illumination and noise level data. We investigate the importance of ambient sensing in combination with inertial sensors to address the class imbalance problem of human activity context recognition. Like some of the works reviewed above, we combined inertial and ambient sensing to recognize human activity contexts using deep convolutional neural networks for automatic feature extraction, fully connected neural networks, and sliding window with overlapping as signal segmentation algorithm for activity context classification.

## 3. Methods and Materials

Activity context recognition based on multi-class classification algorithms requires labeled training datasets, in which training samples belong to known classes or categories. The samples representing these classes are usually not evenly distributed. We have classes with a higher number of samples forming the majority classes and those with very few samples making up the minority classes. With this skewed dataset, classification algorithms will typically over-classify the majority classes because of their higher prior probability, whereas the minority classes are misclassified due to their very low prior probability. To address the problems, our method takes a data-centric approach and combines DCNN, inertial, and ambient data augmentation. The data-centric approach provides additional sensing signals from the ambient sensors in addition to the traditional sensing signals from inertial sensors. This section describes the architecture of the proposed system and the structure of the DCNN.

**Figure 2:**
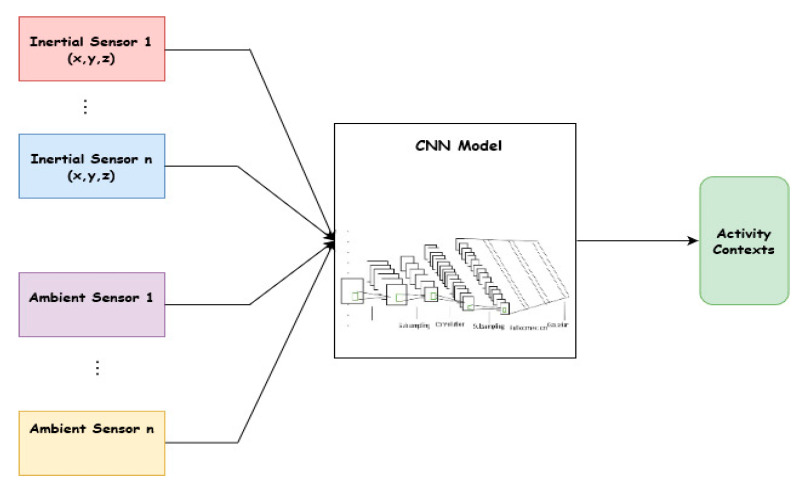
Inertial and ambient sensing model.

The overview of the method is illustrated in Figure 2, which shows sensing data from both inertial and ambient sensors as inputs to the DCNN. In the literature, three categories of techniques have been applied to address the class imbalance problem. These approaches are classified into three types, namely data-based approaches, algorithm-based approaches, and the hybrid approach that combines both [33]. We adopted this approach to enhance the recognition accuracy of the minority classes. This requires an additional dataset to augment the original data collected from inertial sensors. The process involves collecting labeled data using sound (microphone and speakers) and light sensors. In total, signals from 3 inertial and 2 ambient sensors were analyzed. First, labeled data was collected from only inertial sensors. The second step involved collecting labeled data using all the 5 sensors. These sensors and the corresponding signals represent 9 classes. In the next section, we describe these sensors.

### 3.1. Inertial Sensing

**Figure 3:**
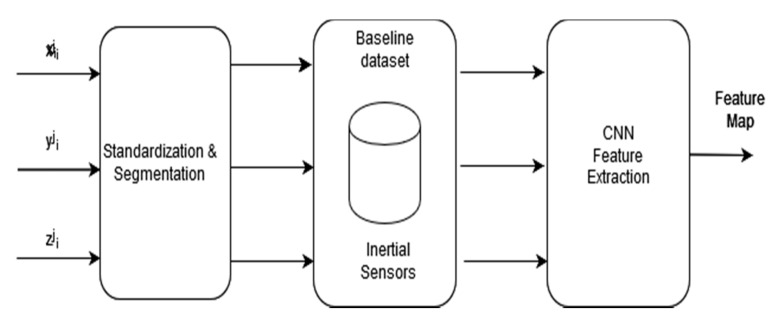
The baseline model with inertial sensing data.

Inertial sensors are the most used sensors for activity context recognition [2,3,4,5,6,7,8,9,10]. In contrast to vision-based systems, inertial sensing does not pose many privacy issues and is available on most smartphones. The baseline model developed in our system uses inertial sensor signals, like several other existing systems. However, instead of using data from the accelerometer and gyroscope as other works have done, we added a magnetometer as an additional sensor. Figure 3 illustrates the feature extraction model for the raw inertial sensing data. The following are the inertial sensors used by the proposed system.

**Figure 4:**
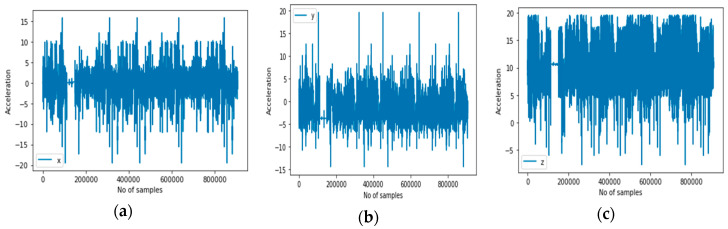
3-D accelerometer signals representing all activity contexts. (**a**–**c**) respectively are the x, y, and z axes of the accelerometer sensor.

**Figure 5:**
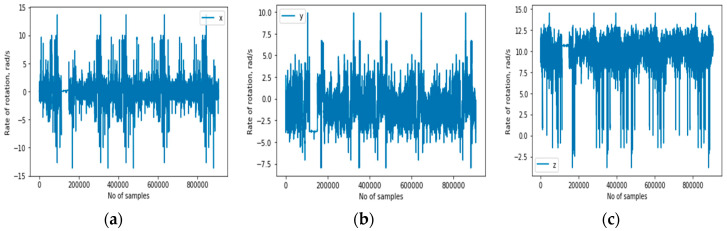
3-D gyroscope signals representing all activity contexts. (**a**–**c**) respectively are the x, y, and z axes of the gyroscope sensor.

**Figure 6:**
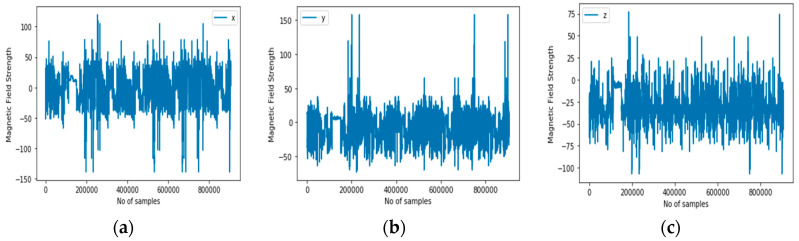
3-D magnetometer signals representing all activity contexts. (**a**–**c**) respectively are the x, y, and z axes of the magnetometer sensor.

(a) Accelerometer: It is a motion sensor that measures the acceleration in m/s^2^, along three axes. The measurement is the rate of change of velocity of the object. Figure 4 illustrates the accelerometer sensor’s representation of the signals for the activity context classes in 3D.
(b) Gyroscope: It is a sensor that measures the orientation and angular velocity of an object. A gyroscope is an advanced form of accelerometer that is about to capture the tilt and lateral orientation of an object, whereas the accelerometer only measures the change in linear velocity. Figure 5 illustrates the gyroscope sensor’s representation of the signals for the activity context classes in 3D.
(c) Magnetic sensor (magnetometer): This position sensor measures magnetic field strength and directions. Such magnetic field results from the movement of charges or electrons. It is generally used to measure the induction. It is an important component of aircraft but now is being used as one of those sensors for detecting human activities. This is because several mobile devices now come with magnetometers. Normally, it has been used to detect the orientation of the mobile phone relative to the Earth’s magnetic north. Figure 6 illustrates the magnetometer sensor’s representation of the signals for the activity context classes in 3D.

### 3.2. Ambient Sensing

Several approaches addressing class imbalance rely on oversampling of the minority classes by synthetically generating additional samples [2]. Some other works downsample the majority classes [34]. Our approach follows the oversampling method, but rather than generating synthetic sample data, we used additional data collected from two ambient sensors.

**Figure 7:**
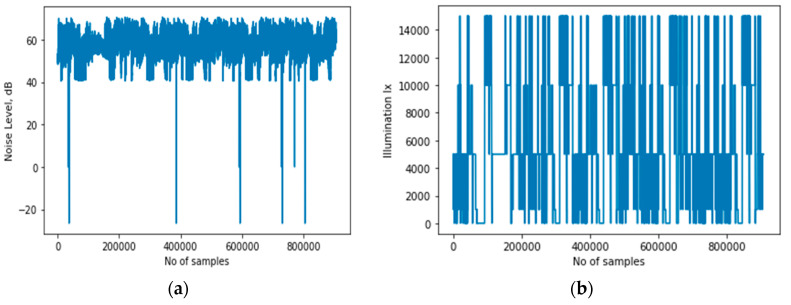
1D audio (**a**) and light (**b**).

(a) Sound sensor: consisting of a microphone and speaker. Modern smartphones usually have a pair of built-in speakers and a microphone. These can be used to recognize human activities and other ambient conditions, such as noise level. While the microphone receives the ultrasound signal, the speaker transmits the signals.
(b) Light sensor: generates an output signal that indicates the intensity of light by measuring the radiant energy that exists in a narrow range of frequencies, and which ranges from infrared, through visible light up to ultraviolet light spectrum. Figure 7 illustrates the environment noise and illumination representation of the activity context classes.

**Figure 8:**
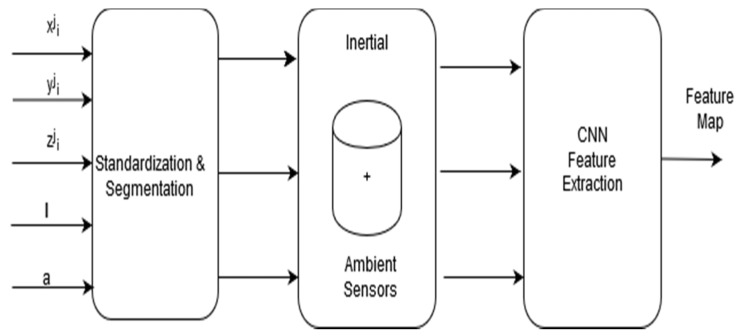
Inertial and ambient sensing model.

Figure 8 illustrates the feature extraction model for both ambient and inertial sensing data, where *l* and a represent the illumination and audio sensing signals as inputs in addition to the inertial sensing data, denoted by $x_{i}^{j},\;y_{i}^{j},\;and\;z_{i}^{j}$, i.e., axes of the inertial sensors.

### 3.3. The Architecture and Structure of the Proposed System 

**Figure 9:**
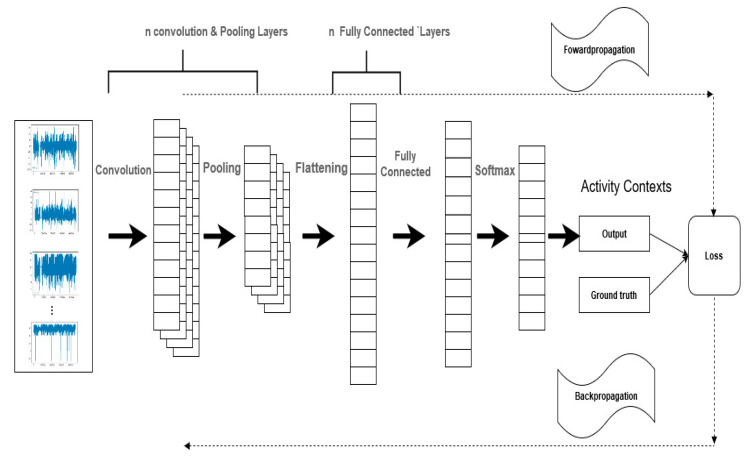
Architecture of the proposed system.

Figure 7 illustrates the architecture of the proposed DCNN model. It consists of 3 types of layers: the convolutional and pooling layers; the flattened and the fully connected layer; and the output layer. Note that, in Figure 9, where n denotes the number of layers. The value of n is determined later, in Section 4. In the first layer, the sensors signals are fed as inputs to 3 convolutional layers stacked with corresponding max-pooling layers and Rectified Linear Unit (ReLU) activation function [20,22]. Automatic feature extraction is executed at this layer. The resulting feature maps represent the activity context classes. We arrived at the number of convolution layers after a set of experiments to determine an optimal number with the highest recognition accuracy, as reported in Section 4.3.1. The second component of the architecture consists of flattened and fully connected layers. The flattened layer accepts the feature maps from the previous layer (max-pooling layer) and converts the feature maps to a single column vector that is then fed to the fully connected layer. The fully connected layer performs the classification process. The final layer is the output layer, i.e., Softmax layer, that receives the outputs of the fully connected layer and computes the probability distribution of each class [16,17]. The recognition model was trained to minimize cross-entropy errors with L2 regularization and dropout probability to prevent overfitting [17,22,34]. We optimized the model’s hyperparameters using Adam optimization, and backpropagation to compute the gradient of the loss function [35].

#### 3.3.1. Raw Data Pre-processing

The two important preprocessing techniques used are data standardization and segmentation using sliding windows with overlapping [36].

(a) Data Standardization

To minimize bias [16,17], we standardized the data samples by subtracting the mean from the original value and then dividing the result by its standard deviation. Both the 3D inertial signals and 1D ambient signals were standardized using Equation (1).

(1) $$\frac{x-\bar{x}}{\sigma }$$

(b) Segmentation

**Figure 10:**
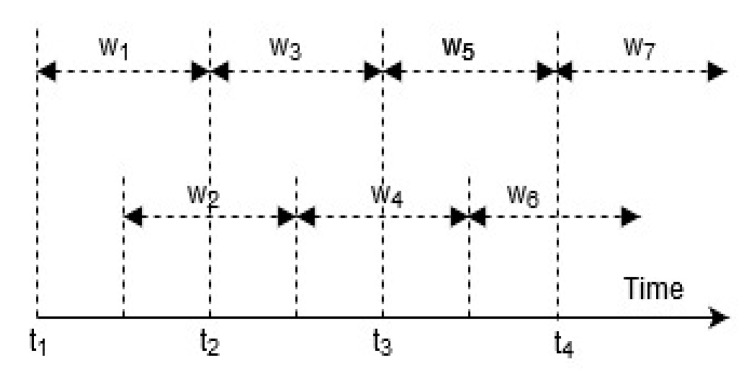
Sliding window with 50% overlaps.

Following the standardization of the input signals, a temporal sliding window algorithm was applied [4]. In this process, the input data is split into data segments of fixed intervals of samples called “windows”. Each window contains a small part of the sensor signal [3,32]. As illustrated by Figure 10, each window is 50% “*overlapped*” to form the next window, preserving a proportion of the previously sampled signal data overlapping the start of the next sample [3,27,35].

### 3.3.2. The Proposed Deep Convolutional Neural Networks Structure

**Figure 11:**
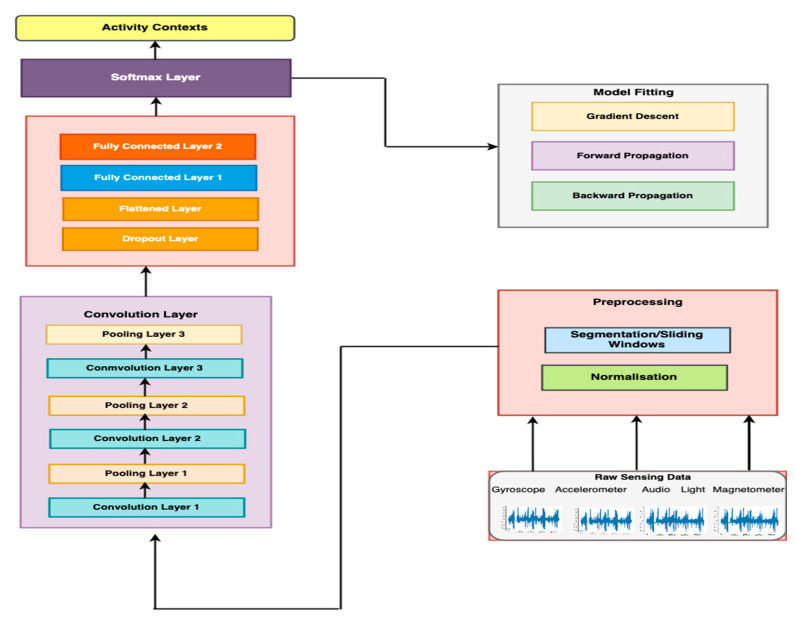
Deep convolutional neural network (DCNN) activity context recognition processes.

In this section, we describe the structure of the DCNN model, as illustrated in Figure 11. 

(a) Convolutional layer

The convolutional layer is responsible for the automatic feature extraction process [12]. We assume that $x_{i}^{a}=\left[x_{1},\ldots ,x_{N}\right]$ are inputs from the sensors, where a represents the axis of the sensors. Depending on the number of convolutional layers, the feature map of the lth convolutional layer is computed using Equation (2).

(2) $$z_{i\;}^{l,j}=\sigma \left(\sum_{k=1}^{K}w_{k}^{j}x_{i+k-1}^{l-1,j}+b_{j}^{l}\right),$$

where $w_{k}^{l,j}$ and $b_{j}^{l}$ are the weight and bias of the j-th term of the l-th layer. $x_{i+k-1}^{l,j}$ is the input patch, l is the index of the current layer, and σ is the activation function. K represents the size of the filter/kernel. The activation function $\;a_{j}^{l}=\;\sigma (z_{i\;}^{l,j})$ introduces non-linearity to the CNN layer for detecting the discriminative features of the raw sensing data.

(b) Pooling Layer

The pooling layer is responsible for the downsampling operation of the generated feature maps of the convolutional layer [9,22]. We used max-pooling operation [5], which outputs the maximum value from every patch of inputs (Equation (3)).

(3) $$f_{i}^{l,j}=max_{s\in S}\left(z_{i*T+s\;}^{l,\;j}\right),$$

where S is the pooling size, and its stride is denoted by T. $z_{i*T+s\;}^{l,\;j}$ is the value of the i−th node in layer l.

(c) Fully connected layer

Following the convolutional and max-pooling operations, the feature maps produced by the last convolutional and max-pooling layer are then flattened into a one-dimensional (1D) vector of features $f^{l\;}=\left[f_{1},\ldots ,f_{l}\right]$, where l is the number of nodes in the last pooling layer.

This is then fed as input to the fully connected layer. The output of the pooling layer is illustrated in Equation (5).

(4) $$f_{i}^{l}=\sum_{j}^{}w_{ij}^{l-1}\left(\sigma (f_{i}^{l-1}\right)+b_{i}^{l-1}\;\;),$$

where σ is the ReLU activation function, $w_{ij}^{l-1}$ is the weight connecting the i−th unit in layer l−1 and the j−th  unit in layer  l, and $b_{i}^{l-1}$ is the bias.

(d) Softmax layer

The output of the fully connected layer is fed to the softmax layer to produce the inferred class. The softmax layer uses the softmax function to compute the probability distribution of each class. If the activation function of *j*-th output neuron is:

(5) $$a_{j}^{l}=\frac{e^{x_{J}^{L}}}{\sum_{K}e^{Z_{J}^{L}}},$$

then the probability distribution is computed using Equation (7):

(6) $$P\left(c|f\right)=argmax\;\frac{e^{f^{l-1}}w^{L}b^{L}}{\sum_{k=1}^{N}e^{f^{l-1}}w_{k}},$$

where N is the total number of classes or the number of neurons in the output layer, and $a_{j}^{l}$ is the activation function of output node j.

For each CNN layer and fully connected layer, we applied a ReLU activation function [11,12,22], as in Equation (4), applied pointwise to the outputs of their respective CNN layer.

(7) ReLU=f(x)=max(0,x).

(e) Regularization

Regularization allows the model to generalize to test or unseen data [22]. To prevent overfitting due to large weights, which is a very common problem in deep neural networks, a dropout layer was added to the network. Adding dropout means randomly and temporarily dropping some nodes, including all their incoming and outgoing connections [22,34]. Besides the dropout probability, we also applied weight decay [17,22], as illustrated in Equation (9).

(8) $$w_{i}\to \overset{´}{w_{i}}=w_{i}-\alpha \lambda w_{i}-\alpha \frac{\partial L}{\partial w_{i}}.$$

(f) Backpropagation

In each iteration, following the forward propagation (performed by Equations (2)–(7)), the loss error is computed. The error is the difference between the predicted class and the ground truth using the loss function L, by applying the Adam optimization [22,35]. The backpropagation iteration executes until a stopping criterion, i.e., epoch has been satisfied, using the chain rule of derivative [16,17,22].

In the fully connected layer, the gradient descent is computed using the classical partial derivatives and chain rules.

(9) $$\frac{\partial L}{\partial w_{i,j}^{l}}=y_{i}^{l}\frac{\partial L}{\partial x_{j}^{l+1}},$$

where L is the categorical cross-entropy loss function that defines multiclass s logarithmic loss by comparing the distributions of the predictions with those of the ground truths setting the probability of the ground truth to [0, 1], $y_{i}^{l}=\sigma \left(x_{i}^{l}\right)+b_{i}^{l}$, and σ=Non−linear mapping function. $w_{i,j}^{l}$ is the weight connecting node $n_{i}^{l}$ in the network layer l, the network node $n_{i}^{l+1}$ at layer l+1, and the total number of input nodes $n_{i}^{l+1}$ is $x_{j}^{l+1}$.

(10) $$L=-\sum_{i}^{T}{\hat{y}}_{i}log\left(softmax\left(y_{i}\right)\right),$$

where softmax is the function (Equation (7)) that outputs the probability distributions of each class. 

In the 3 convolution layers of the model, the backpropagation is executed by computing the gradients of the layer’s respective weights using Equation (12) based on the chain rule:

(11) $$\frac{\partial L}{\partial w_{a,b}}=\sum_{i=1}^{N-M-1}\frac{\partial L}{\partial x_{i,j}^{l}}y_{i+a}^{l-1}.$$

$y_{i+a}^{l-1}=\sigma \left(x_{i+a}^{l-1}\right)+b_{i}^{l-1}$, and σ=Nonlinear mapping function  of the convolution layer. $\frac{\partial L}{\partial x_{i,j}^{l}}$ = $\frac{\partial L}{\partial x_{i,j}^{l}}\sigma^{l}\left(x_{i,j}^{l}\right)\;$. This process is repeated until the maximum epoch (the stopping criteria) is reached.

## 4. Experiments and Evaluation Results

In this section, we present our experimental setup, including the analysis of the obtained results.

### 4.1. Dataset and Experimental Setup

The data used in this experiment was obtained from inertial and ambient sensors. We provided a full description of the process involved in our data gathering from the smartphone’s built-in sensors in Reference [3,37], where we used conventional machine learning algorithms and handcrafted statistical feature extraction processes. In the experiments conducted in this article, we used two datasets to train the developed models. We describe the datasets in the next sections. 

#### 4.1.1. The Inertial Dataset

**Figure 12:**
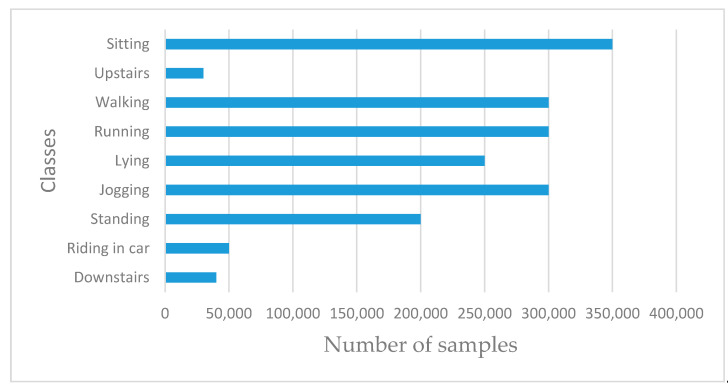
Distribution of classes by number of samples.

Unlike most existing works that used the 3D accelerometer dataset [8,9,12,16,17], we collected data from additional inertial sensors, namely gyroscope and magnetic sensors. The dataset was collected using our mobile app that was developed for the data collection process [3]. Figure 12 shows the distribution of classes, including the number of samples for each class. Three of the classes have a significantly lower number of samples (“downstairs”, “riding in a car”, and “upstairs”). Details of the data collection process and the mobile app that was developed are presented in Reference [3].

#### 4.1.2. The Inertial and Ambient Dataset

To augment the inertial sensing data in Section 4.1.1 with additional sensing signals, ambient data was collected from two additional sources: audio and light sensors. Whilst the first dataset consists of signal data from 3 inertial sensors, the second dataset consists of data from both inertial and ambient sensors. This is done to allow us to investigate, if any, the importance of ambient sensors for recognizing human activity contexts with imbalanced classes. Like the first dataset, the speaker, loudspeaker, and light sensors were used in tandem with the inertial sensor to collect data representing 9 classes of activity contexts. The audio and the light sensor signals were further processed into the representations of environmental noise and illumination as shown in Table 1.

Besides, we divided each of the two datasets into five sub-datasets, with window lengths of 32, 64, 128, 256, 512, and 1024 samples (approximately 0.75, 1.5, 3, 6, 12, and 24 s, respectively). With the raw inertial and ambient sensors, we performed 17 and 15 channel 1D convolutions. Table 1 shows our experimental setup. Note that part of the pre-processing conducted standardization using Equation (1).

### 4.2. Evaluation Metrics

The metrics used in the context recognition experiments are precision, recall, F-Score, and confusion matrix. These are the most widely used metrics to evaluate context recognition models [3,10,24].

*F-Score* is an often-used metric in information retrieval and natural language processing communities, and it is interpreted as the weighted average of precision (*P*) and recall (*R*). It is a measure of the statistical accuracy of the models given as follows:

F-Score (R, P) = 2*RP/(R + P), (12)

where *Recall* (*R*) is the measure of the ability of a classification model to select instances of a certain class from a dataset. It is the sensitivity of the model defined as:

*R = TP/(TP + FN)*. (13)

*TP* is the number of true positive predictions and *FN* is the number of false-negative predictions.

*Precision (P)*: is the measure of the accuracy if a specific class is predicted; defined as:

*P = TP/(TP + FP)*. (14)

*FP* is the number of false-positive predictions.

*Confusion matrix* is a square matrix of order *n* number of classes used to present detailed results of a multiclass classification problem. The confusion matrix provides a more detailed and fine-grained analysis of both correctly and incorrectly classified classes of the supervised learning-based models. A given element *c_i, j_* of the matrix is the number of instances belonging to class *i*, classified as class *j*. Information about classification errors is also presented by the confusion matrix.

### 4.3. Experiments and Performance Evaluation 

The experimental models were implemented using the Python 3 [38], Keras, and TensorFlow libraries [39]. Two models were implemented. The first model is the baseline model. This model was trained with the first dataset, i.e., th inertial sensing data. The second model was trained using inertial and ambient sensing data. We performed extensive experiments to gain insights into various aspects of deep convolutional neural network-based activity context recognition using ambient and inertial sensors. To train the models, 70% of the dataset was used, while the remaining 30% was used for testing. The experiments are broadly categorized into three types. The first set of experiments investigated the impacts of various hyperparameters on the performance of the system. These experiments were performed to determine the best/optimal values for the parameters to build the final recognition models. In the second set of experiments, first, we investigated the recognition accuracy of the model using inertial sensing data with imbalanced classes. In the third experiment, we evaluated the recognition accuracy of the system when trained with both inertial and ambient sensing data. In the following sections, we provide details of these experiments and present the results.

#### 4.3.1. Hyperparameter Sensitivity Evaluation

Hyperparameter value selection is one of the most difficult parts of training an optimal learning model [22]. It is both an optimization problem (whereby we are looking for the hyper-parameter configuration that generates the lowest validation error) and a generalization problem (whereby we are looking for the configuration of the parameters that reduce estimation bias after optimizing validation performance) [22]. Therefore, the goal of this set of experiments is to carefully choose optimal configurations of the hyperparameters to produce models with not only minimal test error but also with the least bias. In this section, we evaluate the impacts of various sliding windows lengths, decay values, batch sizes, learning rates, dropout probabilities, number of nodes in the fully connected layer, and the number of CNN layers. 

a. Impact of sliding window size on the recognition accuracy

**Figure 13:**
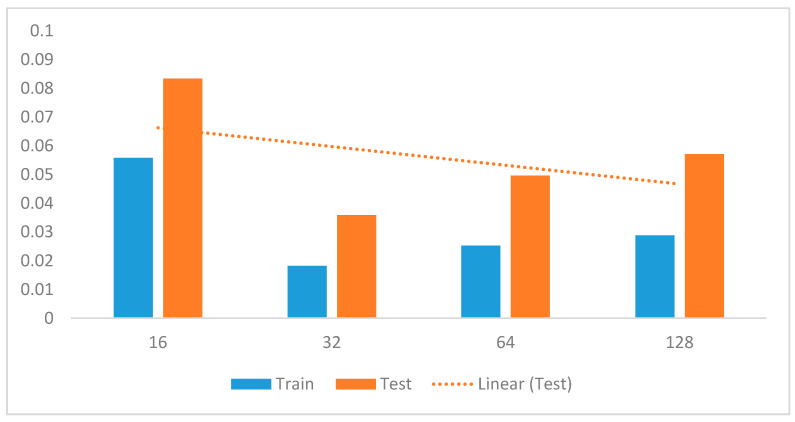
Recognition accuracy with different window sizes overlapping at 50%.

The segmentation of sensing data, especially time-series data, is a crucial pre-processing mechanism. One of the key advantages of segmentation is that it allows us to provide hidden discriminative information in the time series data [3,17,25,36]. The sliding window algorithm with a 50% overlap was used as a data segmentation process. The details of the impact of sliding window sizes from 16 to 128 are summarized in Figure 13, showing the accuracy of the system. The results show that the initial window size of 16 with a 50% overlap produced the highest error, followed by window lengths of 64 and 128 with increasing loss error. Window size 32 has the lowest loss error; thus, it is the optimal value used in subsequent experiments.

b. Influence of pooling size on the model’s accuracy 

**Figure 14:**
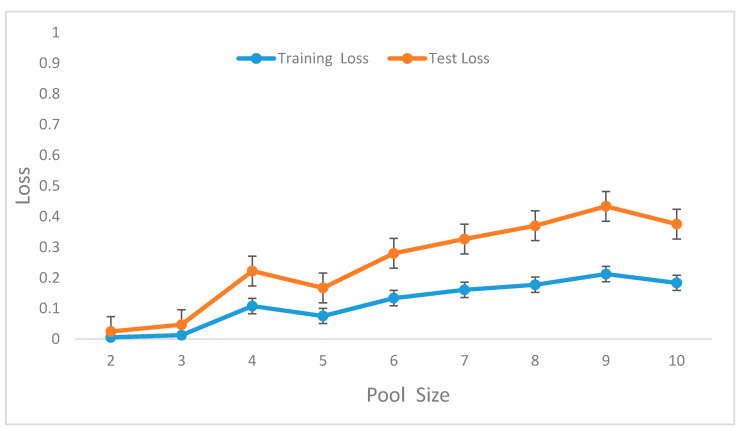
Recognition accuracy of the model for various values of pool size.

We evaluated the impacts of various pooling sizes on the recognition performance of the DCNN model’s configuration. We used 3 convolutional layers, filter size 16, 32, and 64, one fully connected layer with 1024 nodes, sub-sampling factors of 2, 3, and 4, and a final softmax layer for generating the posterior probability of each class. The max-pooling values were increased from 1 to 10 where the max-pooling size of 1 is equivalent to no max-polling process. Figure 14 shows the influence of the various pooling sizes on the accuracy of the model. The best performance was obtained between 3 and 5 although the loss of the pool size of 4 is higher than that of pool size of 5. However, the loss increases from a pool size of 6 to 9.

c. Impact of learning rates on CNN context recognition accuracy

**Figure 15:**
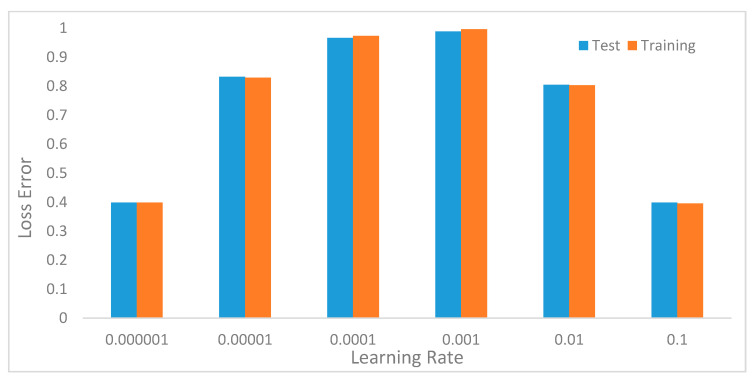
Model recognition accuracy of the model with various values of learning rates.

The initial learning rate is often considered as one of the most important hyperparameters to tune to obtain good model performance. Normally, the values of the learning rate are usually less than 1. Most practitioners rely on a value of 0.01 for standard multi-layer neural networks. To choose the optimal learning rate for our model, we evaluated the $\alpha =\left(10^{-6},\;10^{-1}\;\right)$. Figure 15 shows the performance of the model with various values of the learning rate. The performance improved from $10^{-6}$ with 39.9% accuracy reaching the peak at $10^{-3}$ with 98.8% accuracy. The accuracy started to decline from a learning rate value of 0.01 finally back to 39.9% at learning rate 0.1.

d. Impact of minibatch size

**Figure 16:**
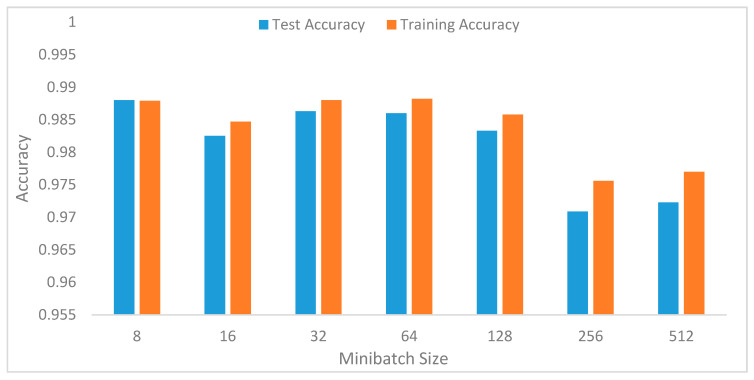
Recognition accuracy of the model with various batch sizes.

The minibatch size controls the accuracy of the estimate of the loss function. It impacts the training process in terms of convergence time and the amount of overfitting [22,40,41]. Smaller batch sizes tend to lead to faster computation, but this requires visiting more examples to compute loss error during the training process. In this experiment, we varied the values of batch sizes from 8 to 512, with increasing values of 8. Figure 16 shows the performance of each batch size. Initially, the performance improves with increasing batch size. However, initially, batch size 8 tends to overfat, but, from a mini-batch size of 16, the model generalizes, but the accuracy was decreased with the increasing number of batch sizes. The best performance was achieved with a batch size of 32 and then dropped from a batch size of 64. The larger minibatch size of course will make greater gradient steps, thus producing poor performance.

e. Impact of decay on CNN context recognition accuracy

**Figure 17:**
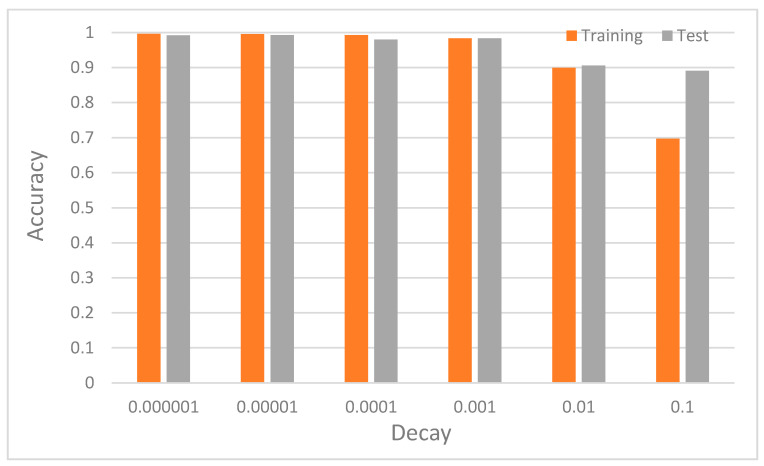
Recognition accuracy of the model with various values of decay.

**Figure 18:**
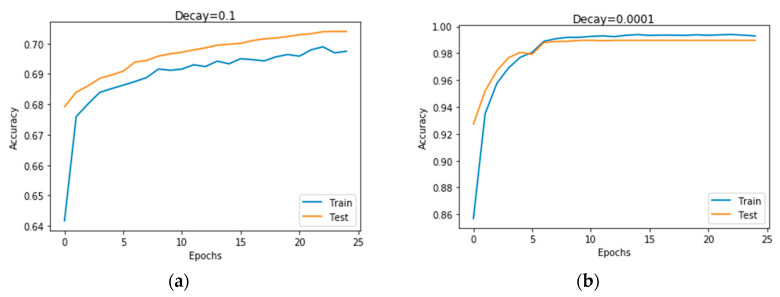
Recognition accuracy of the model with decay values of 0.1 and 0.0001. (**a**) shows that at higher value of 0.1 the model overfits whereas (**b**) shows that at lower values such as 0.0001, the system was able to generalize.

Another important regularization mechanism to eliminate overfitting and improve generalization is weight decay, which is also known as L2 regularization [22]. The learning rate determines how much weight updating steps influence the current value of weights. Weight decay is used to cause the weight to decay exponentially to zero. To ascertain the optimal value of the decay for our DCNN model, we tuned the decay values between $10^{-6}$ to $10^{-1}$. Figure 17 illustrates the impact of each decay value on the performance of the model. The accuracy generally increases from the value of 0.1 and then does not change from 0.0001. The significance of the result is that a further reduction in the decay value does not improve the accuracy of the model. This means that a very small value of decay is required for the model to reach its best recognition performance. Figure 18a,b show the worst performance at 0.1, which demonstrated that, at high values of decay, the model tends to be biased.

f. Impact of dropout on the CNN context recognition accuracy

**Figure 19:**
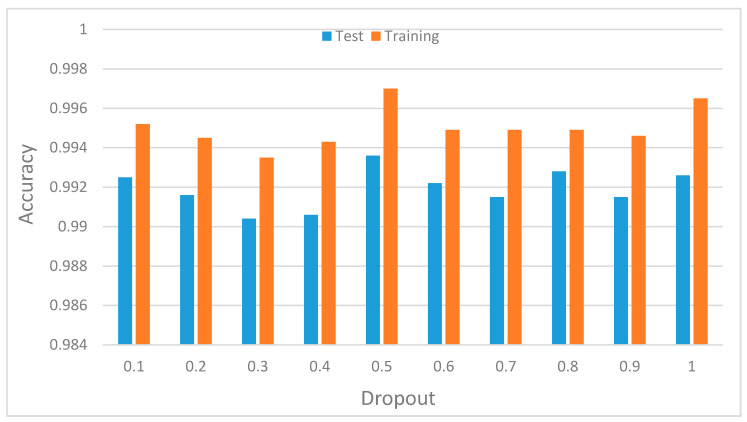
Model recognition accuracy with varying values of dropout probability.

One of the key regularization techniques is dropout as explained in Section 3. The dropout represents the probability of retaining a hidden node in the network. The decision on which nodes to drop is random and node dropping is done independently for each hidden node. Therefore, using the appropriate values for the dropout parameter helps the model to better learn redundant patterns in the time series input features. In the conducted experiment, we varied the values of the dropout parameter (p), while keeping the values of other parameters fixed, i.e., the number of hidden nodes and other parameters were kept constant, just as in other experiments, but only the values of dropout changes. Figure 19 shows the test and training accuracies. Note that the value ranges between 0.1 and 1.0. The best probability is 0.5 reaching 98.8% test accuracy. However, the values of p remain the same for both 0.9 and 1.0 but vary for other values of p. This result shows that, by randomly dropping connections in the hidden layers and applying it in the top fully connected layer, the generalization errors can be reduced, thereby preventing overfitting.

g. Influence of the number of CNN layers context recognition accuracy

**Figure 20:**
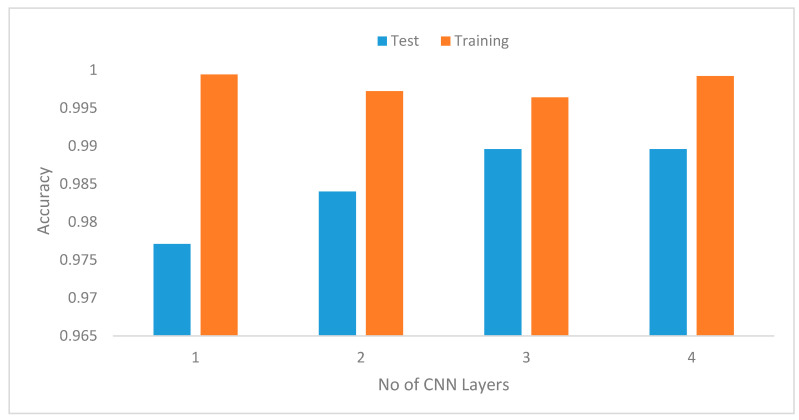
Model recognition accuracy with increasing number of CNN layers.

To determine the number of CNN layers required for the DCNN, we conducted experiments using the same values for other parameters as explained in Section b above, where we varied the values of l from 1 to 4. Figure 20 illustrates the improvement in performance from l=1 to 3, and the accuracy begins to decline from l=4. This result is expected since increasing the number of layers generally is expected to produce better performance. However, in the future, we would like to evaluate the computation cost of increasing the number of CNN layers running on real mobile devices.

h. Impact of the number of nodes in the fully connected layer 

**Figure 21:**
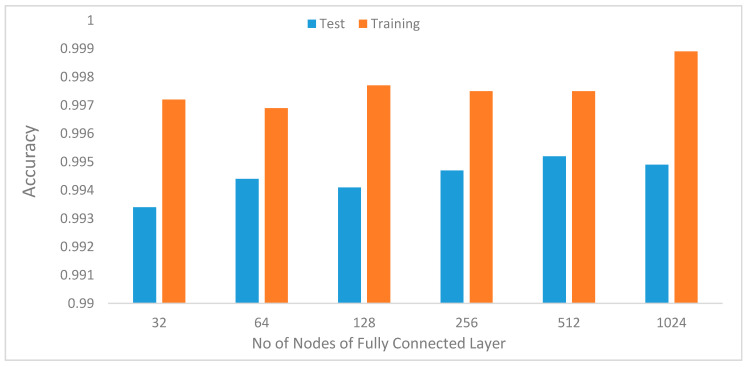
Model recognition accuracy with increasing number of nodes in the fully connected layer.

Another experiment was performed to determine the optimal number of nodes in the fully connected layer. To determine the best value, we varied the values from 32 to 1024. As shown in Figure 21, the performance initially improves from 32 to 64 and declines before peaking at 512. This indicates that we do not need to use many nodes in the fully connected layer to achieve better performance.

### 4.3.2. The Proposed System’s Performance Evaluation

Having analyzed the impact of various hyperparameters on the accuracy of the proposed model, we now investigated the influence of combining data from ambient sensing with inertial sensors. First, we analyzed the results obtained using only inertial sensing data. Secondly, we compared results from inertial only sensing data and those of inertial and ambient sensing data combined. Table 1 illustrates the values of hyperparameters used based on the results of Section 4.3.1.

a. Recognition accuracy using the baseline model using Inertial sensing data

The goal of the experiments in this section was to evaluate the performance of the model when trained with data signals from inertial sensors. The inertial sensors, as explained in Section 4.1, include motion and position sensors, namely accelerometer, gyroscope, and magnetometer. We set the values of our hyperparameters to those optimal values obtained in the hyperparameter sensitivity evaluation, as illustrated in Table 1.

**Figure 22:**
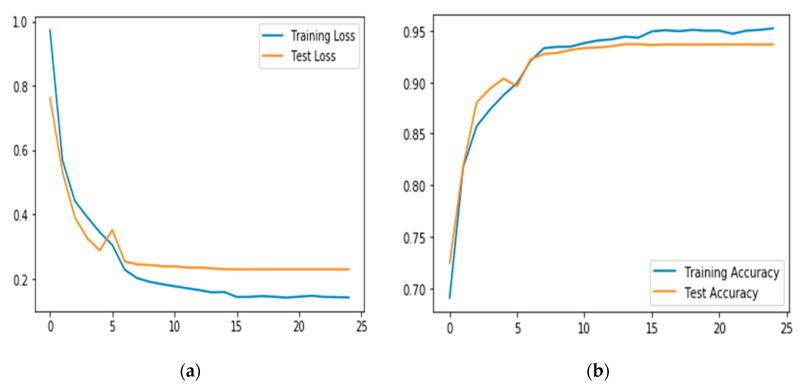
Recognition error/loss (**a**) and recognition accuracy (**b**) of inertial sensing data.

Table 2 represents the confusion matrix of the results obtained showing the class-wise recognition accuracy of the model of each class. Note the performance of those classes with lower accuracy as measured by FScore. As expected, some of the classes have poor recognition performance. The result shows that *climbing*, followed by *climbing downstairs* performed worse than any other class reaching a poor value of 0.56 and 0.67, respectively. Compared to *Running* and *Jogging* classes shared the highest FScore value of 0.98. This result aligns with our initial hypothesis, as reported in literature, that recognition models generally tend to have poor recognition performance when dealing with imbalanced classes and tend to perform well with classes that have a majority number of samples. Besides, Figure 22a,b represents the overall accuracy of the model. The overall performance of the model based on inertial sensing reached up to 93.6% accuracy.

b. Recognition Accuracy using both inertial and ambient sensing data

**Figure 23:**
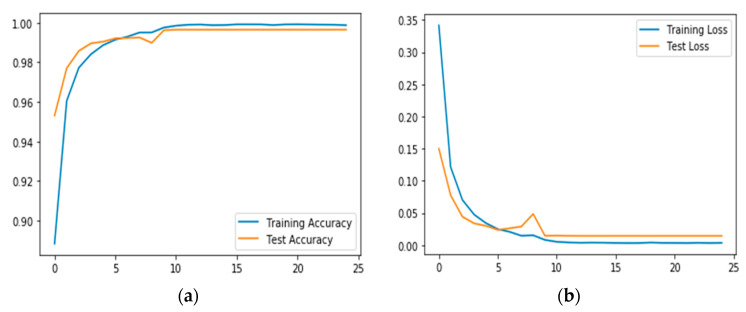
Training accuracy (**a**) and loss performance (**b**) of Inertial + Ambient sensing data.

A key hypothesis of this article was that combining sensing data from both inertial and ambient sensors would produce a better performance of the CNN model. Having evaluated its performance with sensing data from inertial sensors, this section evaluates its performance with both sets of data. We also compared the results with the obtained in a of Section 4.3.2. The results in Table 3 shows the confusion matrix. This shows the performance of the model in terms of recognition accuracy for each activity class. The accuracy of the model reaches up to 98.9% as can be seen in Figure 21. This shows a marginal increase of 5.3 % in recognition accuracy compared to when we used only inertial sensing signals. This improvement is further elaborated in Figure 23a,b, where we compared FScore for each of the activity context classes when using both datasets. The results indicate that models with inertial sensing struggled to recognize certain activity albeit considering good FScore value, for example, in *climbing upstairs* and *sitting* activity contexts. However, the model with both signals produced a far superior performance. Table 2 is the confusion matrix showing the class-wise performance of the model of the new model. As can be seen, the recognition accuracy of those classes in the previous experiment significantly improved. In a of Section 4.3.2, downstairs and upstairs have 0.67 and 0.56, respectively. But in the current experiment, the new model achieved far better performance recognizing these activity contexts with 0.99 FScore value. This improvement is elaborated in Figure 23. For all classes, the model trained with both inertial and ambient data consistently performed better than the model trained with only inertial data. 

## 5. Discussion and Conclusions

Activity context recognition using multichannel, time series inertial sensors have been extensively studied [42]. In this article, we investigated the possibility of using ambient sensing data and deep convolutional neural networks for activity context recognition using a dataset with imbalanced classes. The inertial sensor signals were collected from the gyroscope, accelerometer, and magnetometer. The ambient sensor signals were collected from audio and light sensors, representing environment noise level and illumination, respectively. In our previous work [3,37], we used classical machine learning algorithms with handcrafted features.

In the current work, our goal was to demonstrate that sensing data representing environment noise level and illumination when combined with inertial sensor data to train DCNN models can improve the model’s recognition accuracy. We used the CNN to automatically extract features from the raw inertial and ambient sensing signals. Two DCNN models were implemented and trained. The first model implements the baseline approach, whereas the second model implements the new approach. To evaluate the performance of the proposed models, experiments were designed to compare results obtained from the baseline model and the proposed model trained using inertial and ambient sensing data for multi-class activity context recognition.

In the preliminary experiments, we performed evaluations to select optimal window lengths in the segmentation process using the sliding window with an overlapping algorithm. This experiment informed the decision to use a window length of 32 in the subsequent experiments since this value produced the best recognition accuracy. We then tuned the model’s hyperparameters to determine their optimal values [41]. Hyperparameters, such as learning rates, batch size, decay, dropout, number of CNNs, and the fully connected layers and pool size, as well as the number of nodes in the fully connected layers, were tuned through extensive experiments. Table 1 summarizes the optimal values obtained for these parameters.

The next set of experiments was performed to evaluate the performance of the baseline model. This is where we trained the networks with inertial sensor signals. As illustrated in Figure 12, three classes, namely “Downstairs”, “Riding in a car”, and “Upstairs”, contained fewer samples than other classes. As expected, the classes with fewer samples generated poor recognition performance. The result shows that *climbing upstairs (upstairs*), followed by *climbing downstairs*, performed worse than any other class reaching a poor value of 0.56 and 0.67, respectively. We then used an additional dataset with ambient sensing signals representing environment noise level and illumination from audio and light sensors, respectively. Experimental results confirmed that using these additional signals to augment inertial sensor datasets produced better recognition performance than the baseline model trained with inertial sensor data, with improved global accuracy of 5.3 percent. The results also confirmed significant improvement in the recognition performance of those classes with the least number of samples. Besides, we used various techniques, such as regularization techniques, e.g., L2 regularization (aka weight decay) and dropout, to prevent overfitting of the models. The developed DCNN model shows its capability to automatically extract features from the raw sensing data with better performance compared to the laborious and time-consuming handcrafted features and classical machine learning algorithms used in Reference [3]. The developed DCNN model demonstrates the capability to capture local dependencies of the activity context signals using the correlation of both the inertial and ambient data signals. It also demonstrates that combining signals from ambient sensors produces better recognition performance than using signals from only inertial sensors. In addition, our experimental results demonstrate the influence of various hyperparameters on the eventual DCNN models.

There are limited existing works that have used noise level and illumination to augment inertial sensing data to improve the performance of activity context recognition. One recent work that used inertial and ambient sensing data is the one by Cruciani et al. [13]. The authors evaluated their method using inertial and ambient use cases but did not combine sensing signals of both sensors. For inertial sensing, they achieved 91.98% compared to the work presented in this article, achieved 93.6% accuracy, whereas combination inertial and ambient sensing achieved 98.9% accuracy. Another recent work is the one by Schrader et al. [32]. They used an accelerometer as an inertial sensor combined with a camera and body pressure measurement system. They evaluated the performance of the systems for locomotion and hand gesture activities, achieving accuracies of 0.9 and 0.87, respectively.

In conclusion, in this article, we have demonstrated that with inertial and ambient sensing data, namely environment noise level and illumination, performance of recognition models trained with imbalanced classes can be improved. Experimental evaluations of the implemented models showed performance improvements in accuracy by 5.3% when compared to the baseline model. In addition, extensive parameter tuning experiments were performed to inform the selection of optimal values to build the DCNN models. These results provide valuable insights into the sensitivity of hyperparameters. This article also demonstrates that the DCNN can perform better recognition accuracy with raw inertial and ambient signals without performing a handcrafted feature extraction process than with manually extracted features.

Lastly, one of the key benefits of using ambient sensing is that there is a limited connection to the users in the environment, thus preserving the privacy of individuals [28]. Such independence makes it a better approach for monitoring elderly people’s activities and for other applications, such as intelligent recommendation services. However, one major disadvantage of the current activity context model is that it classifies simple classes; it only recognizes a single context in terms of the activity of the user, i.e., it cannot combine certain contexts, such as location, to predict much semantically meaningful contexts. In the future, we plan to update the system to integrate a semantic model able to combine activity contexts with other ambient contexts, as well as location information and user preferences, to provide much higher level of contextual information. Finally, we will be investigating the computational cost of the model on resource-constrained devices considering the architecture of the model and the number of sensors involved when performing real-time activity context recognition.

## Figures and Tables

**Table 1:** Experiment Parameters.

Hyperparameter	(Ambient + Inertial)	Inertial
**Window size (size of input vectors)**	32	32
**Epochs from**	25	25
**Kernel size**	1 × 3 − 1 × 5	1 × 3 − 1 × 5
**Batch Size**	32	32
**Learning Rate**	0.001	0.001
**Decay**	0.0001	0.0001
**Dropout**	0.5	0.5
**Pooling Size**	3 × 3	3 × 3
**Activation Function**	ReLU (CNN layer), Softmax	ReLU (CNN layer), Softmax
**Input Channels**	17	15
**Fully connected layer (No. of nodes)**	512	512

**Table 2:** Confusion Matrix for Inertial Sensing.

Predicted Class												
Actual Class		1	2	3	4	5	6	7	8	9	FScore	Label
	1	46	0	1	0	0	0	2	0	28	0.67	Downstairs
	2	0	70	0	0	0	1	1	2	2	0.70	Riding in car
	3	0	0	491	0	3	0	0	0	2	0.89	Standing
	4	0	0	0	437	0	6	2	1	4	0.98	Jogging
	5	0	0	5	0	180	0	0	1	1	0.77	Lying
	6	1	0	1	6	0	1397	26	1	12	0.98	Running
	7	2	0	1	3	0	4	362	6	19	0.90	Walking
	8	7	0	6	0	0	9	6	56	40	0.56	Upstairs
	9	4	0	4	0	2	4	7	9	1146	0.94	Sitting

**Table 3:** Confusion Matrix for Inertial + Ambient Sensing.

Predicted Class												
Actual Class		1	2	3	4	5	6	7	8	9	FScore	Label
	1	629	0	2	1	0	0	0	1	2	0.99	Downstairs
	2	0	304	0	0	0	1	0	0	0	1.00	Riding in car
	3	0	0	821	1	3	0	1	0	1	0.99	Standing
	4	0	0	0	2561	0	0	0	0	0	1.00	Jogging
	5	0	0	4	0	136	0	0	0	0	0.97	Lying
	6	0	0	0	3	0	1892	10	1	3	0.99	Running
	7	1	0	1	0	0	1	2697	0	4	1.00	Walking
	8	1	0	0	0	0	1	4	898	4	0.99	Upstairs
	9	1	0	0	0	0	2	3	3	5725	1.00	Sitting
